# The M-current contributes to high threshold membrane potential oscillations in a cell type-specific way in the pedunculopontine nucleus of mice

**DOI:** 10.3389/fncel.2015.00121

**Published:** 2015-04-07

**Authors:** Csilla Bordas, Adrienn Kovacs, Balazs Pal

**Affiliations:** Faculty of Medicine, Department of Physiology, University of DebrecenDebrecen, Hungary

**Keywords:** M-current, neuromodulation, oscillatory activity, spike frequency adaptation, pedunculopontine nucleus

## Abstract

The pedunculopontine nucleus is known as a cholinergic nucleus of the reticular activating system, participating in regulation of sleep and wakefulness. Besides cholinergic neurons, it consists of GABAergic and glutamatergic neurons as well. According to classical and recent studies, more subgroups of neurons were defined. Groups based on the neurotransmitter released by a neuron are not homogenous, but can be further subdivided. The PPN neurons do not only provide cholinergic and non-cholinergic inputs to several subcortical brain areas but they are also targets of cholinergic and other different neuromodulatory actions. Although cholinergic neuromodulation has been already investigated in the nucleus, one of its characteristic targets, the M-type potassium current has not been described yet. Using slice electrophysiology, we provide evidence in the present work that cholinergic neurons possess M-current, whereas GABAergic neurons lack it. The M-current contributes to certain functional differences of cholinergic and GABAergic neurons, as spike frequency adaptation, action potential firing frequency or the amplitude difference of medium afterhyperpolarizations (AHPs). Furthermore, we showed that high threshold membrane potential oscillation with high power, around 20 Hz frequency is a functional property of almost all cholinergic cells, whereas GABAergic neurons have only low amplitude oscillations. Blockade of the M-current abolished the oscillatory activity at 20 Hz, and largely diminished it at other frequencies. Taken together, the M-current seems to be characteristic for PPN cholinergic neurons. It provides a possibility for modulating gamma band activity of these cells, thus contributing to neuromodulatory regulation of the reticular activating system.

## Introduction

The pedunculopontine nucleus (PPN) is known as one of the cholinergic nuclei of the reticular activating system. Its proposed roles are the modulation of sleep and wakefulness, contribution to attention and sensory gating, and coordination of movements (Garcia-Rill, 1991; Reese et al., 1995; Maloney et al., 1999; Jenkinson et al., 2009; Garcia-Rill et al., 2011).

Although the PPN is considered as a cholinergic nucleus, it is composed of non-cholinergic (i.e., GABAergic and glutamatergic) neurons, as well. Neurons of this nucleus are grouped due to their functional or morphological characteristics. According to the classical grouping based on electrophysiological properties, three types of neurons were distinguished. However, none of the functional groups are clearly related to morphological subgroups (Kang and Kitai, 1990; Leonard and Llinás, 1994; Steriade and McCarley, 2005).

More recently, *in vivo* recordings revealed functional subgroups according to the relationship of single unit activity to global brain states (Mena-Segovia et al., 2008; Ros et al., 2010). Briefly, the majority of cholinergic neurons fire during cortical up states and increase their activity in parallel with cortical gamma activity. In contrast, a fraction of cholinergic cells fire in time with the cortical down state, and do not fire synchronously with gamma activity (Mena-Segovia et al., 2008). The non-cholinergic neurons (including GABAergic and glutamatergic neurons) have at least three functional subgroups, as tonically firing, irregular and quiescent neurons were identified, which fire in different phases of cortical activity (Ros et al., 2010).

According to the functional heterogeneity of the PPN neurons, to the best of our knowledge, no single electrophysiological marker has been identified yet, which can clearly distinguish between cholinergic, GABAergic or glutamatergic PPN neurons.

The M-type potassium current is a slowly activating, non-inactivating voltage-gated potassium current, which is activated at subthreshold potentials. Its name came from the classically proposed pathway of its inhibition, i.e., muscarinic acetylcholine receptor activation closes the channel (Brown and Adams, 1980; Marrion, 1997; Delmas and Brown, 2005; Brown and Passmore, 2009; Hernandez et al., 2009). However, the muscarinic acetylcholine receptor is not the only G-protein coupled receptor which is capable of modulating this conductance: the bradykinin, histamine, angiotensin, metabotropic glutamate, adrenergic, purinergic, substance P or opiate receptors can also close it (Marrion, 1997; Delmas and Brown, 2005; Brown and Passmore, 2009). Furthermore, receptors altering intracellular calcium or cAMP levels can also effectively modulate this channel (Chambard and Ashmore, 2005; Linley et al., 2012).

Classical functions of the M-current are the contribution to the medium and slow afterhyperpolarizations (AHPs) of the action potentials (mAHP, sAHP; e.g., Madison and Nicoll, 1984; Storm, 1989; Koyama and Appel, 2006; Tzingounis and Nicoll, 2008; Tzingounis et al., 2010; Mateos-Aparicio et al., 2014), the spike frequency adaptation (e.g., Madison and Nicoll, 1984; Nigro et al., 2014), shaping of the action potential firing properties, setting the resting membrane potential (e.g., Madison and Nicoll, 1984; Koyama and Appel, 2006; Navarro-López et al., 2009; Guan et al., 2011; Nigro et al., 2014) and regulation of presynaptic functions (e.g., Huang and Trussell, 2011). Furthermore, M-current contributes to neuronal membrane potential oscillations at a characteristic resonance frequency. Injecting sinus wave current with a continuously increasing frequency to hippocampal pyramidal cells, the M-current dependent maximal resonance frequency was around 6–8 Hz (e.g., Hu et al., 2002).

PPN neurons are known to possess high threshold membrane potential oscillations. These oscillations are activated by depolarization exceeding −25 mV. Oscillations largely depend on N- and P/Q type calcium channels and dendrotoxin-sensitive potassium channels (Kezunovic et al., 2011; Simon et al., 2011). Carbachol leads to the temporary inhibition of oscillatory activity, followed by the return of oscillatory waves with a higher frequency (Kezunovic et al., 2013; Garcia-Rill et al., 2014).

In the present work, we showed that the vast majority of PPN cholinergic neurons possess M-current, whereas GABAergic neurons completely lack it. The presence or absence of M-current contributes to certain electrophysiological differences between cholinergic and GABAergic neurons, as amplitude of medium afterhyperpolarization, spike frequency adaptation and firing frequency. It was also shown that the majority of cholinergic neurons have high amplitude oscillatory activity with a peak around 20 Hz, while GABAergic neurons have an oscillatory activity with much smaller amplitudes. The blockade of the M-current completely abolished the oscillations of cholinergic neurons around 20 Hz and reduced it at other frequencies, indicating that the M-current has a significant contribution to the oscillatory activity of cholinergic PPN neurons.

## Materials and Methods

### Animals, Preparation, Recordings

Animal experiments were conducted in accordance with the appropriate international and Hungarian laws and institutional guidelines on the care of research animals. The experimental protocols were approved by the Committee of Animal Research of the University of Debrecen. 9–16 days old mice expressing tdTomato fluorescent proteins in a GAD2- (*n* = 11) or ChAT-dependent way (*n* = 19) were used for the experiments. The homozygous floxed-stop-tdTomato (Madisen et al., 2010; JAX mice accession number: 007905), GAD2-cre (Taniguchi et al., 2011; JAX number: 010802) and ChAT-cre (http://www.informatics.jax.org/reference/J:114556; JAX number: 006410) mouse lines were purchased from Jackson Laboratories (Bar Harbor, ME, USA) and crossed in the animal house of the Department of Physiology. For obtaining preliminary data, 9–16 days old C3H (*n* = 7) and Bl6 (*n* = 5) mice were also used from both sexes. After decapitation of the animal, the brain was removed, and 200 μm-thick coronal midbrain slices were prepared in ice-cold low Na aCSF using a Microm HM 650V vibratome (Microm International GmbH, Walldorf, Germany). Brain slices were visualized with a Zeiss Axioskop microscope (Carl Zeiss AG, Oberkochen, Germany). The microscope was equipped with a fluorescent imaging system (Till Photonics GmbH, Gräfeling, Germany) containing a xenon bulb-based Polychrome V light source, a CCD camera (SensiCam, PCO AG, Kelheim, Germany), an imaging control unit (ICU), and the Till Vision software (version 4.0.1.3). Patch pipettes with 5 MΩ pipette resistance were pulled from borosilicate glass, and filled with a solution detailed in the subsection “Solutions, chemicals”. Whole-cell patch-clamp recordings were performed using an Axopatch 200A amplifier (Molecular Devices, Union City, CA, USA), either in voltage- or current-clamp modes. Data acquisition was achieved using the Clampex 10.0 software (Molecular Devices, Union City, CA, USA), while data analysis was performed using the Clampfit 10.0 (Molecular Devices) program.

For recording M-current, the neurons were washed with 1 μM tetrodotoxin (TTX; Alomone Labs, Jerusalem, Israel), and held on −20 mV holding potential, and 1-s-long repolarizing steps were applied from −30 to −60 mV (Brown and Adams, 1980). The pharmacological isolation of the M-current was achieved by using its specific blocker, 20 μM XE991 (10, 10-*bis*(4-Pyridinylmethyl)-9(10*H*)-anthracenone dihydrochloride; Tocris Cookson Ltd., Bristol, UK; Wang et al., 2000).

Current-clamp recordings were performed by using 10 pA depolarizing current square pulses. Adaptation index was calculated from traces where at least 8 action potentials were found, by using the following formula: AI = 1 − (F_last_/F_initial_), where F_last_ is the average frequency of the last 2 action potentials, and F_initial_ is the average frequency of the first three (Nigro et al., 2014). Fast afterhyperpolarization (fAHP) was determined as the maximal negative potential change compared to the action potential threshold within 50 ms after the action potential spike, medium afterhyperpolarization (mAHP) was measured as the amplitude at 100 ms after the spike, and slow afterhyperpolarization (sAHP) was defined as the amplitude at 300 ms after the spike (see e.g., Koyama and Appel, 2006). For detection of oscillatory activity, 2-s-long ramp protocol was used with 800 pA maximal amplitude in the presence of TTX. All experiments were performed on room temperature (Kezunovic et al., 2011, 2013).

All data represent mean ± SEM. Statistical significance was determined using Student’s *t*-test; the level of significance was *p* < 0.05.

### Visualization of the Biocytin Labeled Neurons

The neurons were filled with biocytin during the electrophysiological recordings. The slices accommodating the filled neurons were fixed overnight (4% paraformaldehyde in 0.1 M phosphate buffer; pH 7.4; 4°C). Permeabilization was achieved in Tris buffered saline (in mM, Tris base, 8; Trisma HCl, 42; NaCl, 150; pH 7.4) supplemented with 0.1% Triton X-100 and 10% bovine serum (60 min). The slices were incubated in phosphate buffer containing streptavidin-conjugated Alexa488 (1:300; Molecular Probes Inc., Eugene, OR, USA) for 90 min. The cells were visualized using a Zeiss LSM 510 confocal microscope (Carl Zeiss AG). When wild type C3H or Bl6 mice were used, cholinergic cells were identified with anti-choline acetyltransferase labeling (1:75; Millipore, Temecula, CA, USA) and 1:1000 Texas Red rabbit-anti-goat secondary antibody (Vector Laboratories Inc., Burlingame, CA, USA). Data obtained from both ChAT-positive and -negative neurons were considered as preliminary data.

### Solutions, Chemicals

Experiments were performed in an artificial cerebrospinal fluid (aCSF) of the following composition (in mM): NaCl, 125; KCl, 2.5; NaHCO_3_, 26; glucose, 10; NaH_2_PO_4_, 1.25; CaCl_2_, 2; MgCl_2_, 1; myo-inositol, 3; ascorbic acid, 0.5; and sodium-pyruvate, 2. For the slice preparation, ice-cold, low sodium aCSF was used, where 100 mM NaCl was replaced by sucrose (130 mM) and glycerol (60 mM; low Na aCSF). All chemicals were purchased from Sigma (St. Louis, MO, USA), unless stated otherwise. The composition of the pipette solution was (in mM): K-gluconate, 120; NaCl, 5; 4-(2-hydroxyethyl)-1- piperazineethanesulfonic acid (HEPES), 10; EGTA, 2; CaCl_2_, 0.1; Mg-ATP, 5; Na_3_-GTP, 0.3; Na_2_-phosphocreatinine, 10; biocytin, 8. For certain experiments, 1 μM tetrodotoxin (TTX; Alomone Labs, Jerusalem, Israel) was used to block fast voltage-gated Na^+^ currents and action potential firing of neurons, and 20 μM XE991 (10, 10-*bis*(4-Pyridinylmethyl)-9(10*H*)-anthracenone dihydrochloride; Tocris Cookson Ltd., Bristol, UK; Wang et al., 2000), a specific blocker of the M-current was applied for the pharmacological isolation of this current.

## Results

**Figure 1:**
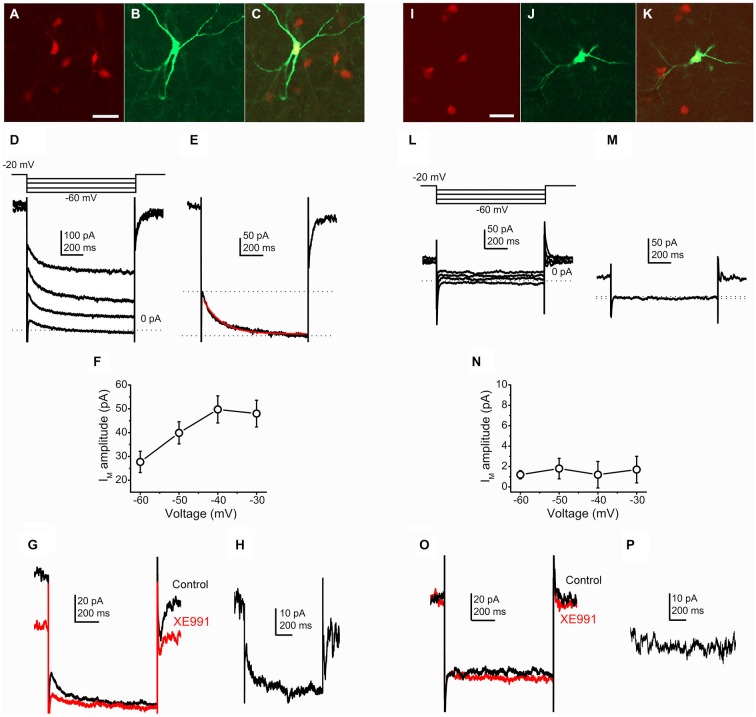
**Cholinergic neurons possess M-current, whereas GABAergic neurons lack it. (A–C)** Identification of a cholinergic neuron. Scale bar = 50 μm. **(A)** ChAT-dependent tdTomato expression (red). **(B)** Biocytin labeling of a recorded neuron (green). **(C)** Merged image. **(D)** Current traces from a cholinergic neuron elicited by the voltage protocol at the top of the panel. **(E)** Current trace at −40 mV repolarizing step. Dotted lines indicate the instantaneous (upper dotted line) and the steady state (lower dotted line) current components. The M-current was determined as the difference of these current components. The red trace indicates the fitting of the declining phase of the current (see text). **(F)** Voltage-dependence of the M-current amplitude (*X* axis: amplitudes of repolarizing current steps). **(G)** Pharmacological identification of the M-current (black = control; red = 20 μM XE991). **(H)** The XE991-sensitive current; calculated by the digital subtraction of the control and XE991-resistant current traces. The clear difference of XE991-sensitive current amplitudes recorded on −20 and −40 mV also represent the presence of M-current. **(I–K)** Identification of a GABAergic neuron. **(I)** GAD2-dependent tdTomato expression (red). **(J)** Biocytin labeling of a recorded neuron (green). **(K)** Merged image. **(L)** Current traces from a GABAergic neuron elicited by the same voltage protocol as on panel **(D). (M)** Current trace at −40 mV repolarizing step. Note that there is almost no difference between the instantaneous and steady state currents. **(N)** None of the repolarizing steps elicited M-current. **(O)** Currents from GABAergic neurons did not show XE991-sensitivity. **(P)** Digital subtraction of current traces under control conditions and in the presence of XE991. The lack of difference of XE991-sensitive current amplitudes recorded on −20 and −40 mV indicates that M-current was not recorded.

In order to detect M-current of different types of PPN neurons, mice expressing tdTomato fluorescent protein in a GAD2- or ChAT-promoter-dependent way were used. The identified tdTomato-expressing neurons were filled with biocytin, and later recovered; and confocal images were taken in order to assess their location and document their GAD2- or ChAT-positivity (Figures 1A–C,I–K).

The M-current, as a non-inactivating potassium current was identified either by using the M-current relaxation protocol or defined as the current component sensitive to its specific blocker, XE991. With the M-current relaxation protocol, stepping down from −20 mV leads to the closure of channels responsible for M-current. The M-current amplitude was determined as the difference of the instantaneous and steady-state current components (Figure 1E).

When recordings were obtained from genetically identified cholinergic neurons, stepping back from −20 mV to more negative voltages, different amplitudes of M-current were revealed (Figures 1D,F). In accordance with data obtained from other structures (Brown and Adams, 1980; Shah et al., 2002; Koyama and Appel, 2006), the maximal amplitude was recorded after stepping back to −40 mV (−49.7 ± 5.1 pA; *n* = 23). However, this average was decreased by two cases when M-current was not detected. Out of these two neurons, the average M-current amplitude was −53.9 ± 4.8 pA, ranging between −25 and −160 pA.

The relaxation kinetics was fitted with a single exponential function (Figure 1E). At the −40 mV voltage step, relaxation of the observed current took place with the decay time constant of 162.8 ± 19.2 ms. The decay time constant showed less pronounced dependence from repolarizing voltage steps. However, the average of the decay tau at −60 mV was 150.8 ± 26.3 ms, which had a tendency of increase when the repolarizing voltage steps were smaller (161.1 ± 14.8 at −50 mV and 162.7 ± 17 ms at −30 mV).

The M-current was further characterized by using its selective blocker, XE991 (20 μM). Washing the slices with this drug, the holding current at −20 mV and the difference between the instantaneous and steady state current compounds at the negative voltage steps were also decreased. The difference of the control and XE991-resistant currents was used as an alternative method for calculation of M-current (*n* = 11). The decrease of the holding current at −20 mV was −39.7 ± 7.5 pA in average on cholinergic neurons, ranging from −19 to −65 pA (Figures 1G,H). In contrast with the cholinergic PPN neurons, GABAergic neurons did not possess a current similar to the M-current (Figures 1L,M). Using the same analysis procedure for measuring M-current amplitude, minimal amplitudes could be recorded in all cases (−1.27 ± 1.29 pA at −40 mV; ranging from +8 to −7 pA; *n* = 13; Figure 1N).

When XE991 was applied, almost no changes were observed in the recorded currents. The holding current at −20 mV had an average change of −3.33 ± 1.8 (ranging between 0 and −6 pA; *n* = 8; Figures 1O,P).

We performed experiments on wild type Bl6 and C3H mice in order to reveal possible differences between mouse strains and to exclude bias caused by expression of tdTomato or cre recombinase. In these cases, the cholinergic and non-cholinergic nature of patched neurons was identified by *post hoc* ChAT-labeling. The amplitude of the M-current of ChAT-positive neurons, calculated as the difference of the instantaneous and steady state current components at −40 mV, was 64.7 ± 17.6 pA. The M-current amplitude, calculated as the XE991-sensitive component at −20 mV holding potential was 60.3 ± 14.8 pA (*n* = 4). Currents of non-cholinergic neurons, calculated in the same way as on cholinergic neurons were either 4.06 ± 2.64 or 2.8 ± 1.65 pA (*n* = 5). Similar to the experiments on Bl6 strain, all ChAT-positive neurons from C3H mice possessed M-current with an amplitude of 48.8 ± 10.1 pA at −40 mV (or 37.75 ± 5.7 pA as XE991-sensitive current at −20 mV; *n* = 6), whereas non-cholinergic neurons did not have M-current (1.66 ± 0.8 pA at −40 mV; −0.2 ± 0.48 at −20 mV as the XE991-sensitive component; *n* = 7). No statistically significant differences were revealed between cholinergic neurons from transgenic and wild type mouse strains, and non-cholinergic and GABAergic neurons from different strains also did not differ significantly.

We concluded that the majority of the genetically identified cholinergic neurons (21 from 23) possess M-current, whereas none of the GABAergic cells displayed this current (*n* = 13).

In the further experiments, we aimed to assess whether the presence or absence of M-current is responsible for certain differences between cholinergic and GABAergic neurons.

**Figure 2:**
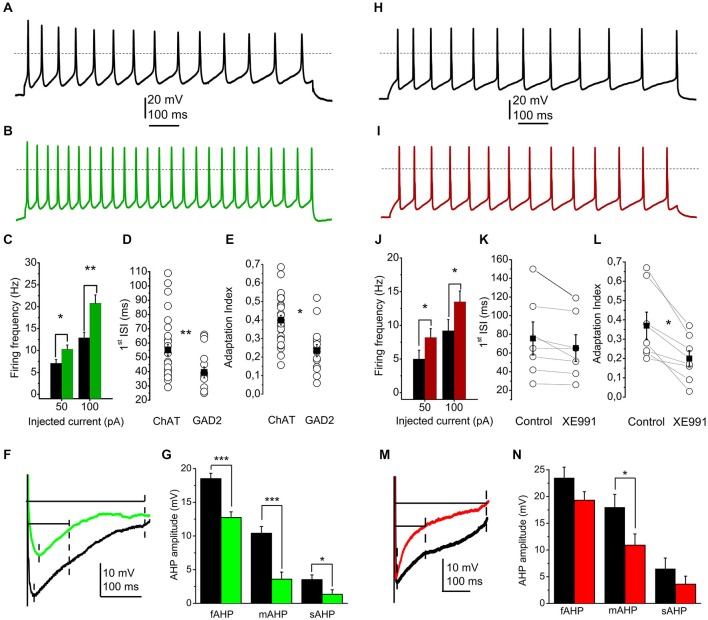
**The presence or absence of the M-current contributes to the electrophysiological differences between cholinergic and GABAergic neurons. (A)** Train of action potentials recorded from a cholinergic neuron, elicited by 100 pA current injection (black). **(B)** Train of action potentials from a GABAergic neuron, elicited by the same stimulus (green). **(C–E)** Statistical comparison of electrophysiological parameters of cholinergic (black) and GABAergic (green) neurons. **(C)** Current injections elicit higher frequency of action potential firing from GABAergic neurons. **(D)** Cell type dependence of the first interspike interval (hollow circles: individual data; black squares: average ± SEM). **(E)** Cell type-dependent changes of the adaptation index (see text; hollow circles: individual data; black squares: average ± SEM). **(F,G)** Cell type dependence of the amplitudes of afterhyperpolarizations (AHPs). **(F)** Voltage traces from a cholinergic (black) and a GABAergic (green) neuron. Dashed lines indicate the points where the fast, medium and slow AHPs were determined. **(G)** Statistical comparison of fast (fAHP), medium (mAHP) and slow AHPs (sAHP). Black: cholinergic; green: GABAergic. **(H)** Train of action potentials recorded from another cholinergic neuron, recorded under control conditions (black). **(I)** Train of action potentials recorded from another cholinergic neuron, recorded in the presence of 20 μM XE991 (red). **(J–L)** Statistical comparison of electrophysiological parameters of cholinergic neurons under control conditions (black) and with 20 μM XE991 (red). **(J)** Current injections elicit higher frequency of action potential firing in the presence of XE991. **(K)** Changes of the first interspike interval by application of XE991 (hollow circles: individual data; black squares: average ± SEM). **(L)** changes of the adaptation index with application of XE991 (see text; hollow circles: individual data; black squares: average ± SEM). **(M,N)** Effect of XE991 on the amplitudes of AHPs. **(M)** Voltage traces from a cholinergic neuron under control conditions (black) and in the presence of XE991 (red). Dashed lines indicate the points where the fast, medium and slow AHPs were determined. **(N)** Statistical comparison of fast (fAHP), medium (mAHP) and slow AHPs (sAHP). Black: control; red: XE991.

In order to achieve this, 28 cholinergic and 17 GABAergic neurons were patched. Parameters likely depending on M-current were investigated, as firing frequency, first interspike interval and adaptation index of an action potential train or amplitudes of fast, medium and slow AHPs. Using current-clamp mode of the whole-cell technique, 1-s-long square pulse current injections were applied with an amplitude of 50 and 100 pA, while the resting membrane potential was set to −60 mV (Figures 2A,B).

We found that injection of 50 or 100 pA current elicited action potential trains with 7.1 ± 0.83 Hz and 12.89 ± 1.28 Hz frequency on cholinergic neurons, whereas 10.28 ± 0.95 and 20.81 ± 1.89 Hz was recorded from GABAergic neurons, respectively. Differences were significant (*p* = 0.01 and 0.001; Figure 2C). In accordance with these data, the first interspike interval of action potential trains elicited by 100 pA current injection also showed marked differences between cholinergic and GABAergic neurons (55.2 ± 4.2 ms for cholinergic and 39.4 ± 3.8 ms for GABAergic neurons, *p* = 0.007; Figure 2D). The adaptation index (AI), as the frequency decrease of action potential trains is also known as a functional characteristic influenced by the M-current (Madison and Nicoll, 1984; Nigro et al., 2014). Cholinergic neurons showed strong adaptation (AI = 0.4 ± 0.02; ranging from 0.15 to 0.68), whereas GABAergic neurons have significantly lower AI (0.23 ± 0.03; between 0.05 and 0.52; *p* = 0.0002; Figure 2E).

Medium and slow AHPs are also known to be influenced by the M-current (Madison and Nicoll, 1984; Storm, 1989; Koyama and Appel, 2006; Tzingounis and Nicoll, 2008; Tzingounis et al., 2010; Mateos-Aparicio et al., 2014). Although fast, medium and slow AHPs could not always been clearly distinguished, we measured the maximal amplitude of the AHP within 50 ms after the action potential spike as fast, the amplitude at 100 ms after the spike as medium, and the amplitude at 300 ms after the spike as slow afterhyperpolarization. The amplitudes were measured as differences of data points between the action potential threshold and the point described above (Figure 2F). We found that all AHPs were significantly larger in cholinergic neurons than GABAergic. Fast AHP was −18.5 ± 0.8 mV in cholinergic and −12.7 ± 0.8 in GABAergic neurons (*p* = 0.0002), medium AHP was −10.4 ± 1 and −3.6 ± 1.1 mV (*p* = 0.0001), whereas slow afterhyperpolarization was 3.52 ± 0.7 and 1.34 ± 0.68 mV, respectively (*p* = 0.016; Figure 2G).

Resting membrane potentials of cholinergic and GABAergic neurons were also compared, but statistically significant differences were not revealed (The resting membrane potential was −56.48 ± 1 mV for cholinergic and −56.72 mV ± 0.83 mV for GABAergic neurons; *p* = 0.44).

In order to assess whether the M-current contributes to the observed differences between cell types, we applied XE991 on 7 cholinergic and 5 GABAergic neurons and recorded the parameters above (Figures 2H,I).

On cholinergic neurons, blockade of M-current led to an increase of action potential frequency to the same current injection (5.1 ± 1.3 Hz under control conditions and 8.2 ± 1.3 Hz in the presence of XE991 at 50 pA current injection; and 9.2 ± 1.6 vs. 13.5 ± 1.6 Hz at 100 pA; *p* = 0.05 and 0.04; Figure 2J).

The first interspike interval was also compared under control conditions and with XE991. Although there was a tendency of decrease, the differences did not prove to be statistically significant (75.6 ± 17.7 ms under control conditions and 65.4 ± 14.4 ms in the presence of XE991; *p* = 0.31; Figure 2K).

The adaptation index always showed a marked decrease when XE991 was applied. Under control conditions, it was 0.37 ± 0.07, whereas after application of XE991 it decreased to 0.2 ± 0.04 (*p* = 0.033; Figure 2L).

The blockade of M-current led to numerical decrease of the afterhyperpolarization amplitudes. However, only the decrease of the medium afterhyperpolarization was statistically significant (−18 ± 2.4 mV in control and −10.9 ± 2.1 mV with XE991; *p* = 0.035; Figures 2M,N).

The resting membrane potential showed numerical depolarization after the application of XE991, but the difference was not significant (−56.71 ± 2.68 mV in control and −52.18 ± 3.7 mV after application of XE991; *p* = 0.17).

Application of XE991 did not result significant changes in the investigated electrophysiological parameters of the GABAergic neurons (*n* = 5). The first interspike interval of this population of GABAergic neurons was 39.1 ± 8.3 ms under control conditions and 41.5 ± 8.4 ms in the presence of XE991, and the adaptation index was 0.18 ± 0.04 in aCSF and 0.17 ± 0.05 with XE991. Similarly, no changes of the different phases of afterhyperpolarization (fAHP: 13.4 ± 3 mV in control and 12.8 ± 2.5 mV with XE991; mAHP: 8.2 ± 2.2 vs. 8 ± 2 mV; sAHP: 2.2 ± 1.2 vs. 3 ± 0.8 mV) and resting membrane potential was revealed (−58.1 ± 1.5 mV in control and −57.6 ± 1.8 mV with XE991).

In order to reveal the contribution of the M-current to the electrophysiological differences between cholinergic and GABAergic neurons, the investigated parameters (firing frequency with 50 and 100 pA current injections, adaptation index and the amplitude of AHPs) were compared between GABAergic neurons under control conditions and cholinergic neurons during treatment with XE991. Interestingly, when blocking M-current of cholinergic neurons, the adaptation index and firing frequency with 50 pA depolarizing steps did not differ significantly between the two groups (although when comparing these parameters of neuronal groups before XE991 treatment of the cholinergic neurons, significant differences were revealed). However, the firing frequency with 100 pA depolarizing step and amplitudes of fast, medium and slow AHPs remained significantly different.

Our data indicate that the M-current of the cholinergic neurons seems to have an important role in differences between cholinergic and GABAergic neurons in the spike frequency adaptation and firing frequency. In contrast, although this current has a contribution to afterhyperpolarization, it is not the only current determining the revealed differences between the two neuronal populations.

In the next series of experiments, high threshold membrane potential oscillations were assessed. These membrane potential changes are largely TTX-resistant, and depend on P/Q- and N-type calcium channels and potassium channels. Oscillations had larger amplitude if current ramp pulses were used to elicit them instead of square pulses (Kezunovic et al., 2011, 2013; Garcia-Rill et al., 2014).

**Figure 3:**
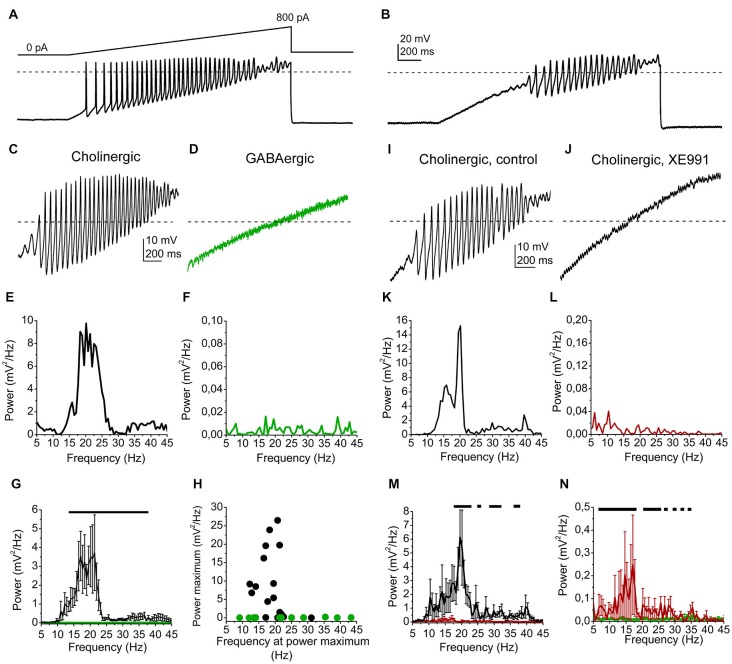
**High threshold membrane potential oscillations are affected by the M-current. (A)** Representative voltage trace from a cholinergic cell elicited by a ramp of depolarizing current injection under control conditions. **(B)** Voltage trace from the same neuron in the presence of TTX. **(C)** High threshold oscillation recorded from a cholinergic neuron in the presence of TTX. **(D)** High threshold oscillation recorded from a GABAergic neuron in the presence of TTX. **(E)** Power spectrum of the oscillatory activity of the cholinergic neuron shown on panel **(C). (F)** Power spectrum of the oscillatory activity of the GABAergic neuron shown on panel **(D). (G)** Statistical summary of the power spectra of all recorded neurons (average ± SEM). Black: cholinergic, green: GABAergic. The black line indicates the frequency range where significant difference was found between datasets obtained from cholinergic and GABAergic neurons. **(H)** Power peaks plotted against the frequencies at the power maximum (black: cholinergic; green: GABAergic neurons). **(I)** High threshold oscillation recorded from another cholinergic neuron in the presence of TTX. **(J)** Records from the same neuron in the presence of 20 μM XE991. **(K)** Power spectrum of the oscillatory activity of the cholinergic neuron shown on panel **(I). (L)** Power spectrum of the oscillatory activity with XE991, shown on panel **(J). (M)** Statistical summary of the power spectra of all cholinergic neurons under control conditions (black) and with XE991 (red; average ± SEM). **(N)** Comparison of power spectra of cholinergic neurons in the presence of XE991 (red) and GABAergic neurons (green; average ± SEM). Black lines of panels **(M,N)** indicate the frequency ranges where statistical differences were found between the two examined populations of data. Dashed lines of panels **(A–D)** and **(I,J)** indicate 0 mV.

In order to elicit these oscillations, 1 s long current ramp injections were used with a maximal amplitude of 800 pA (Figure 3A). Adding 1 μM TTX, action potentials were blocked and only high threshold oscillations were recorded (Figure 3B). Oscillations fell in a frequency range of 10–45 Hz.

We found that most of the cholinergic neurons had high power oscillations, whereas none of the GABAergic neurons had it (Figures 3C–F). A wide range of the power spectra of GABAergic and cholinergic oscillatory activities between 13 and 38 Hz proved to differ significantly (*p* = 0.05 − 0.0001; Figure 3G). The power peak of the oscillations was below 0.5 mV^2^/Hz in 4 cases from 17 cholinergic neurons, whereas it never exceeded the value above in the case of 11 GABAergic neurons. The average peak power of cholinergic neurons was 8.97 ± 2.17 mV^2^/Hz, whereas it was significantly smaller on GABAergic neurons (0.06 ± 0.02 mV^2^/Hz; *p* = 0.001). The oscillation frequency at the power maximum was 19.9 ± 1.07 Hz (ranging from 12.2 to 31.1 Hz) for the cholinergic neurons, whereas it was 23.93 ± 3.49 Hz for the GABAergic neurons (ranging from 9.1 to 39.6 Hz; *p* = 0.06; Figure 3H).

Oscillations of cholinergic neurons were tested in the presence of the M-current blocker XE991, which largely reduced the power of them (Figures 3I–L). Several points of power spectra showed significant differences in the range of 18–23, 28–32 and 37–40 Hz between the records under control conditions and during XE991-treatment (*p* = 0.05 − 0.01; Figure 3M).

Although oscillations were significantly reduced by XE991, the power spectrum with XE991 did not become identical with the one from GABAergic neurons. Oscillation power was significantly larger in the range of 6–19 and 21–24 Hz. Interestingly, the 20 Hz oscillations were almost eliminated by the M-current blocker XE991 (Figure 3N).

## Discussion

In the present project we showed that most of the cholinergic neurons of the PPN possess M-current, whereas all investigated GABAergic neurons lacked it. The M-current of the cholinergic neurons contributes to certain functional differences of cholinergic and GABAergic neurons, as differences in firing frequency, spike frequency adaptation or the amplitude of medium and slow AHPs. Notably, besides these findings, high power membrane potential oscillations of the cholinergic neurons are also modulated by the M-current, as its blockade diminishes its power at all frequencies; fully abolishing it at 20 Hz.

Although the PPN is known as a cholinergic nucleus of the reticular activating system, it is composed of GABAergic and glutamatergic neurons, as well (Garcia-Rill, 1991; Reese et al., 1995; Maloney et al., 1999; Jenkinson et al., 2009; Garcia-Rill et al., 2011). Furthermore, out of the neurotransmitters they release, PPN neurons display a significant level of heterogeneity and can be sorted according to more morphological and functional markers; as neurochemical markers, cellular electrophysiological properties or relationship to global brain states (Kang and Kitai, 1990; Garcia-Rill, 1991; Leonard and Llinás, 1994; Datta and Siwek, 2002; Steriade and McCarley, 2005; Mena-Segovia et al., 2008, 2009; Ros et al., 2010; Garcia-Rill et al., 2011, 2014; Martinez-Gonzalez et al., 2011). GABAergic and cholinergic neurons have different distribution in the rostral and caudal part of the PPN. In the rostral part, GABAergic neurons dominate, whereas cholinergic and GABAergic neurons have a similar number in the caudal PPN. When the whole PPN is considered, the number of the cholinergic neurons is roughly the half of the GABAergic ones (Mena-Segovia et al., 2009). Out of neurotransmitters released and enzymes synthesizing them, neurochemical markers of PPN cells were also revealed: distribution of calcium-binding proteins as calbindin, calretinin and parvalbumine have different distribution in different cell types in different locations of the nucleus (Martinez-Gonzalez et al., 2011).

Here we reveal a potential functional marker with which cholinergic cells can be identified with a higher probability, as 91% of the cholinergic neurons had M-current, but none of the GAD65-positive GABAergic neurons possessed it. Similarly, with our experimental arrangement, oscillations with a power exceeding 0.3 mV^2^/Hz are a feasible functional marker of the cholinergic neurons, because 82% of all cholinergic neurons had this oscillation, where such amplitude was never detected on GABAergic ones.

According to the animal models we used, glutamatergic PPN neurons were not investigated. However, according to our preliminary experiments where cholinergic neurons were identified with *post hoc* ChAT immunohistochemistry, neurons identified as non-cholinergic ones also lacked M-current and high power oscillations. Although the proportion of glutamatergic neurons from this population is uncertain, one can hypothesize that glutamatergic neurons also lack M-current and high power oscillations.

The M-current is known to be present in wide areas of the brain (reviewed by Brown and Passmore, 2009), including brainstem structures (Kharkovets et al., 2000; Koyama and Appel, 2006; Hansen et al., 2008; Navarro-López et al., 2009). It is known as a slowly activating, non-inactivating voltage-gated potassium channel, which is modulated by several metabotropic receptors. According to the classical description, it is closed by the activation of muscarinic acetylcholine receptor; but, however, a long list of receptors can alter its gating properties in different ways (Brown and Adams, 1980; Marrion, 1997; Chambard and Ashmore, 2005; Delmas and Brown, 2005; Hernandez et al., 2008; Linley et al., 2012). Gating of M-current can modulate several electrophysiological characters of the neuron, e.g., control of excitability via setting input resistance and resting membrane potential, determination of firing rate to a given current injection, facilitating the spike frequency adaptation and shaping medium and slow AHPs (Koyama and Appel, 2006; Tzingounis and Nicoll, 2008; Navarro-López et al., 2009; Tzingounis et al., 2010; Mateos-Aparicio et al., 2014; Nigro et al., 2014). The presynaptic role of the M-current was also revealed; this conductance can effectively regulate the release properties of cortical synapses and the calyx of Held (Luisi et al., 2009; Huang and Trussell, 2011).

In the present project it was demonstrated that the majority of PPN cholinergic neurons possess M-current, whereas this current was not recorded from GABAergic PPN neurons under the same experimental conditions. M-current recorded from the cortex, hippocampus had much larger amplitude (exceeding 100–200 pA; e.g., Shah et al., 2002; Nigro et al., 2014), but the dopaminergic neurons of the ventral tegmental area (VTA) had an M-current in the same range (65 pA for the VTA and 54 pA for the PPN; Koyama and Appel, 2006).

In this project we revealed differences between cholinergic and GABAergic neurons in firing frequency to a given current injection, first interspike interval, spike frequency adaptation and amplitudes of medium and slow AHPs, to which the contribution of M-current is well established. In order to verify the contribution of the presence or absence of M-current to the differences between cell types, we recorded the same parameters in the presence of the selective M-current blocker XE991. We revealed that the presence or absence of M-current contributes to differences of firing frequency, adaptation index and the amplitude of medium afterhyperpolarization. Comparing parameters of GABAergic neuronal population with cholinergic neurons under XE991 treatment, we showed that the adaptation index and firing frequency at low frequencies failed to display significant difference between the two neuronal groups, but the difference in firing frequency at higher depolarizing steps and in afterhyperpolarization amplitudes remained significant. This indicates that M-current has an important role in determining spike frequency adaptation in cholinergic PPN neurons, but has an only partial contribution to the firing frequency and the morphology of afterhyperpolarization, together with other conductances which also account for the electrophysiological differences between neuronal types of the PPN (see e.g., Kang and Kitai, 1990; Leonard and Llinás, 1994).

Similar to our results, contribution of M-current was shown to the adaptation index, first interspike interval, firing frequency and afterhyperpolarization in different brain structures (Koyama and Appel, 2006; Tzingounis and Nicoll, 2008; Navarro-López et al., 2009; Mateos-Aparicio et al., 2014; Nigro et al., 2014).

The M-current of hippocampal neurons contributes to their resonance activity: if a sinusoidal current injection was applied with an increased frequency, neurons had maximal voltage resonance at theta frequencies in an M-current dependent way (Hu et al., 2002). PPN neurons have a physiologically occurring membrane potential oscillatory activity in the beta-gamma range, elicited by P/Q and N-type calcium channels and potassium channels (Kezunovic et al., 2011; Urbano et al., 2012). This very intriguing mechanism is a target of cholinergic neuromodulation (Kezunovic et al., 2011, 2013). In this work we largely confirmed data of Kezunovic et al. (2011, 2013) about the oscillatory activity of the PPN. However, according to our data, non-cholinergic neurons had very small oscillation power. This difference is likely due to the different experimental approaches. Kezunovic et al. (2011, 2013) performed their experiments on rats and 37°C, whereas we recorded on preparations from mice on room temperature.

We showed that—at least under our experimental arrangement—high power oscillations are the properties of cholinergic neurons, which are largely modulated by the M-current. When XE991 was used for blockade of M-current, diminished oscillatory activity had still significantly larger power at several frequencies than in the case of GABAergic neurons. However, at 20 Hz (which is the oscillatory activity of cholinergic neurons where the power peak can be found) the oscillatory activity of cholinergic neurons was completely abolished.

Unlike the effect of carbachol, using XE991 caused a simple, one-component inhibition of oscillatory activity (Kezunovic et al., 2013). This finding and our results are in accordance with the well-established fact that muscarinic neuromodulation acts on multiple targets, from which the M-current is only one of the players (see e.g., Picciotto et al., 2012). Furthermore, it was shown that the acute blockade of oscillations by carbachol is achieved via the activation of M2 receptor, and M1 receptor is not involved in this action (Kezunovic et al., 2013). Therefore, it seems to be likely that M1-receptor dependent inhibition of the M-current has a minimal role in the phenomenon observed by us. Rather, the M-current dependent modulation of the oscillations might be a different mechanism involving other Gq protein coupled receptors. There are several possible candidates, as M3, mGluR1 and mGluR5, α1 adrenergic or histamine H1 receptors, which are likely located in the PPN, as their presence was shown either with immunohistochemistry or on mRNA level and with functional methods (Khateb et al., 1990; Vilaró et al., 1994; Hou et al., 2002; Zaika et al., 2006; Kőszeghy et al., 2014). All of these receptors –together with angiotensin II-, serotonin- and several peptide-receptors- are capable of inhibiting M-current (e.g., Brown and Passmore, 2009; Oldfield et al., 2009; Filippov and Brown, 2013). It can also be taken into consideration that increase of intracellular calcium level can also block M-current, therefore all effects changing intracellular calcium concentrations might act on the M-current as well (Marrion, 1997; Hernandez et al., 2008; Brown and Passmore, 2009). The sensitivity of M-current to the changes of intracellular calcium is also high: it has an IC50 at 100 nM, i.e., even moderate changes of intracellular calcium can powerfully regulate this conductance. Furthermore, M-current is modulated by other signaling pathways as well. It is stimulated by the increase of intracellular cAMP and the activation of PKA (Selyanko and Brown, 1996; Gamper and Shapiro, 2003; Chambard and Ashmore, 2005; Linley et al., 2012).

As several G-protein coupled receptors can regulate M-current via different signaling pathways, this current might be an effective contributor to several neuromodulatory actions. There are more overlaps of neuromodulatory actions in the PPN. Orexin and ghrelin (Kim et al., 2009), or endocannabinoid and cholinergic actions depolarize the same neuronal population (Kovács et al., 2015). The similar regulation of the M-current might have a role in overlaps of the neuromodulatory effects on PPN neurons.

Besides sending cholinergic fibers, the PPN is also a target of cholinergic inputs from the laterodorsal tegmental nucleus and the contralateral PPN (Semba and Fibiger, 1992). Therefore, cholinergic actions can powerfully regulate this nucleus and its targets. We showed that the presence or absence of M-current contributes to differences between cholinergic and GABAergic neurons. The possible *in vivo* significance of these findings that cholinergic and non-cholinergic (including GABAergic) neurons have different relationships with global brain states. Most cholinergic neurons fire in phase with the “up” states of slow wave sleep and increase their activity with cortical gamma oscillations, whereas non-cholinergic neurons have three distinct morphological subtypes: “quiescent” neurons increase firing rate with cortical activation, “tonic firing” neurons do not seem to have activity in correlation with cortical activation, whereas “irregular firing” neurons can either increase or decrease firing rate with cortical desynchronization (Mena-Segovia et al., 2008; Ros et al., 2010). Altering spike frequency adaptation, firing frequency or oscillatory activity of the cholinergic neurons, but leaving such properties unaffected in GABAergic ones might contribute to the cortical desynchronization and EEG responses characteristic to REM sleep (Kinney et al., 1998), or increased cortical gamma power and decreased spindle amplitude (Valencia et al., 2013) observed after injection of carbachol to the PPN.

Taken together, the M-current seems to be characteristic for the PPN cholinergic neurons, and it is not present on GABAergic cells. Inhibition of M-current diminished membrane potential oscillations, depicting a possible mechanism of neuromodulatory actions on the PPN cholinergic neurons, which has a significant role in regulation of sleep and wakefulness.

## Author Contribution

CB and AK performed and analyzed the experiments. BP performed, designed and analyzed the experiments and wrote the paper.

## Conflict of Interest Statement

The authors declare that the research was conducted in the absence of any commercial or financial relationships that could be construed as a potential conflict of interest.

## Abbreviations

AI: adaptation index
AHP: afterhyperpolarization
cAMP: 3′-5′-cyclic adenosine monophosphate
ChAT: choline acetyltransferase
EEG: electroencephalogram
EGTA: ethylene glycol tetraacetatic acid
fAHP: fast afterhyperpolarization
GABA: *γ*-aminobutyric acid
GAD: glutamate decarboxylase
HEPES: 4-(2-hydroxyethyl)-1-piperazineethanesulfonic acid
mAChR: muscarinic acetylcholine receptor
mAHP: medium afterhyperpolarization
mGluR: metabotropic glutamate receptor
PPN: pedunculopontine nucleus
REM: rapid eye movement
sAHP: slow afterhyperpolarization
TTX: tetrodotoxin
VTA: ventral tegmental area.
